# Relevance of AIF/CypA Lethal Pathway in SH-SY5Y Cells Treated with Staurosporine

**DOI:** 10.3390/ijms23010265

**Published:** 2021-12-27

**Authors:** Mariarosaria Conte, Rosanna Palumbo, Alessandra Monti, Elisabetta Fontana, Angela Nebbioso, Menotti Ruvo, Lucia Altucci, Nunzianna Doti

**Affiliations:** 1 Department of Precision Medicine, University of Campania ‘Luigi Vanvitelli’, Via L. De Crecchio 7, 80138 Naples, Italy; mariarosaria.conte@unicampania.it (M.C.); elisabetta.fontana@unicampania.it (E.F.); angela.nebbioso@unicampania.it (A.N.); lucia.altucci@unicampania.it (L.A.); 2 Institute of Biostructures and Bioimaging (IBB), National Research Council (CNR), Via Mezzocannone 16, 80134 Napoli, Italy; rosanna.palumbo@cnr.it (R.P.); alessandra.monti@ibb.cnr.it (A.M.); menotti.ruvo@unina.it (M.R.); 3 Biogem, Institute of Molecular Biology and Genetics, Via Camporeale Area P.I.P., 83031 Ariano Irpino, Italy

**Keywords:** cyclophilin A (CypA), apoptosis-inducing factor (AIF), human neuroblastoma SH-SY5Y cells, staurosporine-mediated cell death, AIF(370-394) peptide, caspase-3, PARP

## Abstract

The AIF/CypA complex exerts a lethal activity in several rodent models of acute brain injury. Upon formation, it translocates into the nucleus of cells receiving apoptotic stimuli, inducing chromatin condensation, DNA fragmentation, and cell death by a caspase-independent mechanism. Inhibition of this complex in a model of glutamate-induced cell death in HT-22 neuronal cells by an AIF peptide (AIF(370-394)) mimicking the binding site on CypA, restores cell survival and prevents brain injury in neonatal mice undergoing hypoxia-ischemia without apparent toxicity. Here, we explore the effects of the peptide on SH-SY5Y neuroblastoma cells stimulated with staurosporine (STS), a cellular model widely used to study Parkinson’s disease (PD). This will pave the way to understanding the role of the complex and the potential therapeutic efficacy of inhibitors in PD. We find that AIF(370-394) confers resistance to STS-induced apoptosis in SH-SY5Y cells similar to that observed with CypA silencing and that the peptide works on the AIF/CypA translocation pathway and not on caspases activation. These findings suggest that the AIF/CypA complex is a promising target for developing novel therapeutic strategies against PD.

## 1. Introduction

Parkinson’s disease (PD) is a devastating neurodegenerative disorder for which only symptomatic treatments are available. Developing effective therapies against PD is thereby a major need, and advancements in the knowledge of molecular and cellular mechanisms underlying its pathogenesis and/or progression are crucial. However, as human dopaminergic neurons, primary cells from PD patients are difficult to obtain and maintain. Therefore, studies on PD are almost exclusively performed with established neuronal cell models, including the undifferentiated neuroblastoma SH-SY5Y cell line [1]. To induce cellular stress, SH-SY5Y cells can be treated with staurosporine (STS), a protein kinase inhibitor, which provokes cell death through both caspase-dependent and independent pathways [2,3,4]. Indeed, SH-SY5Y cells treated with high concentrations of STS (over 0.5 µM) do not die following a characteristic necrotic phenotype but rather due to oxidative damage. Consistent with this idea, in the presence of high concentrations of STS, caspase inhibition by z-VAD-fmk, a broad-spectrum caspase inhibitor, reduces the apoptotic phenotype but does not inhibit cell death, which instead appears to be due to oxidative damage [5]. Specifically, high concentrations of STS have been shown to increase caspase-3 activity, Poli ADP-ribosio polimerasi (PARP) proteolysis, and morphological changes indicative of apoptosis, within a few hours of treatment [6,7,8]. It has been also demonstrated that STS treatment provides the nuclear translocation of apoptosis-inducing factor (AIF) from the mitochondria to the nucleus, where it exerts a proapoptotic activity [7,9,10].

AIF is a mitochondria-associated flavin-binding protein implicated in electron transport chain functions and reactive oxygen species (ROS) regulation [11,12,13]. However, it is also an important cell death effector in many cellular stress paradigms [14,15,16]. Upon several apoptotic stimuli, which induce outer mitochondrial membrane permeabilization, AIF is released from mitochondria as a truncated form of about ~57 kDa (AIF(Δ1-121), hereafter tAIF), translocating to the nucleus where induces chromatin condensation, DNA degradation, and cell death, through a caspase-independent mechanism [9,15]. Inhibition or down-regulation of AIF provides neuroprotection in vitro and in a variety of different rodent models of acute brain injury induced by cerebral hypoxia/ischemia (HI), arrest-induced brain damage, epileptic seizures, or even brain trauma [17,18,19,20,21]. Moreover, accumulating evidence also suggests that AIF-induced neuronal cell death can be involved in the progression of neurodegenerative diseases such as PD. In agreement with that, a massive nuclear translocation of tAIF has been observed in the ventral mesencephalon of autopsy samples of patients with PD [22]. In addition, its expression changes in the peripheral blood mononuclear cells of these patients [23].

In different cell and rodent models of acute brain injury, the lethal role of AIF is linked to its interaction with cyclophilin A (CypA) [24,25,26]. CypA is a ubiquitously expressed protein belonging to the immunophilin family with a peptidyl-prolyl *cis-trans* isomerase activity [27]. Current studies in animal models and humans have provided evidence of the critical role of CypA in several human diseases [27]. In neurons, CypA has a pro-apoptotic activity following its association with tAIF, because the complex promotes AIF nuclear translocation and/or DNAse activity [21,22]. Gene silencing of CypA indeed provides a significant neuroprotection effect by preventing the nuclear translocation of tAIF [24,25].

We have previously reported an AIF-based CypA-binding peptide named AIF(370-394) able to inhibit the interaction between the two proteins with an IC50 in the low micromolar range [25]. This molecule has been used in several in vitro models to evaluate the role of the AIF/CypA complex in different paradigms of cell death and also as a template for the design of new selective peptidomimetic inhibitors of the complex [28,29,30,31]. AIF(370-394) selectively inhibits the AIF/CypA complex formation, suppresses the glutamate-induced cell death in neuronal cells, and prevents brain injury in neonatal mice following HI [26]. More recently, AIF(370-394) has been used to demonstrate the crucial role of the AIF/CypA complex on myocyte death in arrhythmogenic cardiomyopathy, significantly expanding to other diseases the potential impact of targeting this complex for therapeutic approaches [32]. 

In this scenario, using AIF(370-394) as a prototypical inhibitor, we have investigated the possible crosstalk between the AIF/CypA complex and STS-evoked cell death in SH-SY5Y.

MTT and flow cytometry assays have been used to assess cell viability and apoptosis, whereas the associated molecular mechanism has been assessed by Western blotting (WB) analysis. Moreover, the efficiency and final outcome of using the AIF blocking peptide have been compared to the silencing of CypA. We find that CypA selective targeting confers significant resistance to STS-induced apoptosis in SH-SY5Y cells and that this effect is related to the blocking of CypA/AIF nuclear translocation without affecting caspases activation. The results provide evidence that the AIF/CypA complex is a promising target for the development of combined therapeutic strategies for the treatment of PD.

## 2. Results

### 2.1. Down-Regulation of CypA Protects SH-SY5Y Cells from Death Induced by STS

**Figure 1:**
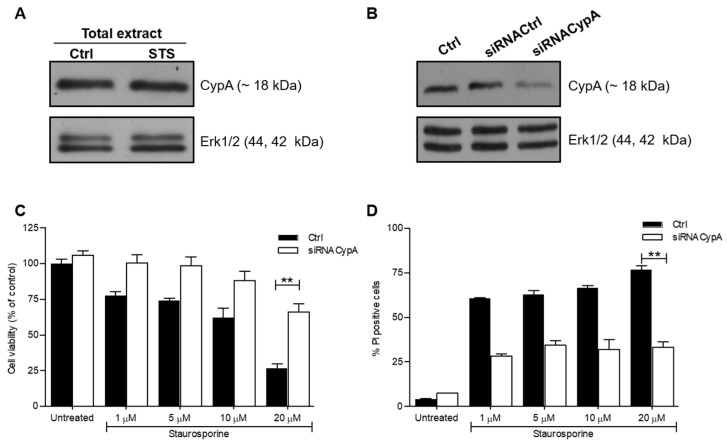
CypA-silencing inhibited STS-induced cell death in SH-SY5Y cells. (**A**) WB evaluation of the expression level of CypA in SH-SY5Y cells untreated and treated for 3 h with 10 μM of STS. (**B**) WB evaluation of CypA expression level after transfection of CypA small interfering RNA (siRNACypA). Erk1/2 proteins were used as a loading control. (**C**) MTT viability assay of SH-SY5Y transfected with siRNACtrl (Ctrl) and siRNACypA exposed to STS for 3 h at the indicated concentrations, (n = 8, ** *p* < 0.01). (**D**) Quantification of flow cytometry results of PI stained SH-SY5Y cells, transfected with siRNACtrl (Ctrl) or with the siRNACypA and treated with STS for 3 h at 10 μM, (n = 8, ** *p* < 0.01).

In order to evaluate the effects of AIF(370-394) on STS-treated SH-SY5Y cells, we first assessed the effects of the down-regulation of endogenous CypA in the cells. CypA was highly expressed in SH-SY5Y and its expression levels increased upon treatment with 10 μM STS, as shown by WB assays of lysates of cells exposed to the drug for 3 h (Figure 1A). The relative densitometric analysis of bands was shown in Supplementary Figure S1A. SH-SY5Y cells were next transiently transfected with a siRNA directed against CypA (siRNACypA) or with an unrelated silencer (siRNACtrl) used as control. As shown in Figure 1B, transfection of the siRNACypA in SH-SY5Y cells provided a decrease of about 60% of CypA levels compared with control groups already after 24 h, as detected by densitometric analysis of WB bands (Supplementary Figure S1B). 

Cell viability 24 h after transfection of the siRNAs was assessed by MTT assays following treatment with different doses of STS (from 1 to 20 μM) for 3 h. In line with previous results [33], STS dose-dependently reduced cell viability reaching a 75% decrease at 20 μM (Figure 1C). Noteworthy, the downregulation of CypA promoted cell proliferation and at the highest concentration of STS (20 μM), cell viability was about 2.8 fold higher (from 25 to 72%, absolute change of about 47%) compared to SH-SY5Y cells treated only with STS (** *p* < 0.01). FACS analyses of apoptotic cells stained with PI were also performed, showing that STS treatment led to a significant percentage of PI positive cells (~60%) already at 1 μM with an increase up to 75% at 20 μM (Figure 1D). Consistent with MTT data, the strong pro-apoptotic effect of STS was neutralized by the downregulation of CypA, which lead to a significant reduction of PI positive cells (from >75% to about 30%) in all conditions tested (Figure 1D). Overall, the results show that CypA is implicated in STS-induced apoptosis in SH-SY5Y cells.

**Figure 2:**
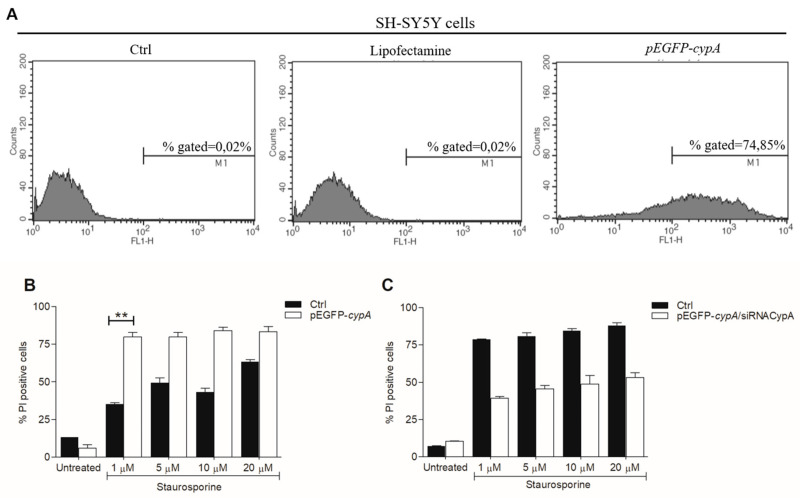
CypA overexpression increases STS-induced cell death in SH-SY5Y cells. (**A**) Assessment of pEGFP-*cypa* plasmid transfection efficiency after 24 h using flow cytometry monitoring the GFP fluorescence. (**B**) Quantification of flow cytometry results of PI stained SH-SY5Y cells, transfected or not (Ctrl) with pEGFP plasmid coding CypA (pEGFP-*cypa*) and treated with STS for 3 h at the indicated concentrations (n = 8, ** *p* < 0.01). (**C**) Quantification of flow cytometry results of PI stained SH-SY5Y cells, co-transfected with pEGFP-*cypa*/siRNACypA or transfected only with pEGFP-*cypA* (Ctrl) and treated with STS for 3 h at the indicated concentrations.

To better illustrate the pro-apoptotic effect of CypA in this experimental paradigm, we also performed experiments in SH-SY5Y cells over-expressing CypA. Cells were transfected with a plasmid coding for CypA fused with the GFP (green fluorescent protein). The efficiency of transfection was assessed by cell sorting monitoring GFP fluorescence. 24 h after the transfection about 75% of cells over-expressed the protein (Figure 2A). Notably, even if the overexpression of CypA induced a negligible cytotoxic effect on SH-SY5Y cells (Supplementary Figure S2), their treatment with STS at 1, 5, 10 and 20 µM for 3 h produced a significant increase of PI positive cells compared to cells not overexpressing CypA (Figure 2B). 

Finally, we co-transfected the cells with the plasmid coding for GFP-CypA and with the siRNACypA. The presence of siRNACypA induced a reduction of CypA expression levels of about 20% at 24 h and the effect increased at 48 and 72 h (Supplementary Figure S3). In line with previous results, the downregulation of CypA at 24 h reduced the percentage of PI stained cells after treatment with STS at all concentrations tested (Figure 2C). Altogether, the results show again that CypA plays a pro-apoptotic role in the cell death of SH-SY5Y induced by STS.

### 2.2. STS-Induced Cell Death Is Counteracted in AIF(370-394)-Treated Cells

Once assessed that CypA plays a role in the neuronal cell loss caused by STS, we used AIF(370-394) to inhibit the formation of the CypA/AIF complex and to evaluate its effect on cell viability compared with that observed following CypA silencing. AIF(370-394) conjugated with a TAT sequence (hereafter AIF(370-394)) was transfected in SH-SY5Y cells with a protocol previously optimized (see Materials and Methods for details). 

**Figure 3:**
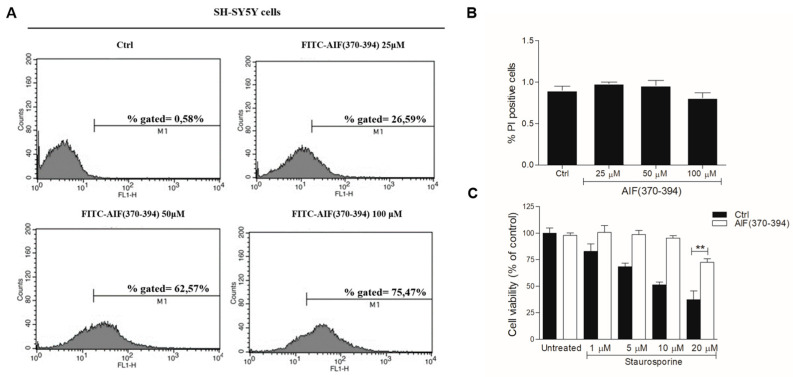
Transfection of the peptide AIF(370-394) protects SH-SY5Y cells from death induced by STS. (**A**) Assessment of AIF(370-394) transfection efficiency after 24 h using flow cytometry and the FITC-conjugated TAT peptide at 25, 50, and 100 μM. (**B**) Evaluation of the cytotoxic effects of FITC-AIF(370-394) by FACS analysis; apoptotic cells were stained with PI. (**C**) MTT viability assay of SH-SY5Y cells transfected with AIF(370-394) exposed to STS for 3 h at the indicated concentrations, (n = 8, ** *p* < 0.01).

The amount of peptide transfected into the cells was determined by FACS analysis using the FITC conjugated peptide at 3 different doses (25, 50, and 100 μM). The average transfection efficiency was about 27, 63, and 75% at 25, 50, and 100 μM, respectively at 24 h (Figure 3A). The effects of peptide transfection on cell viability was explored through FACS analysis by staining the apoptotic cells with the PI dye. Results show that the transfection of the peptide, at all concentrations tested at 24 h, provides no or negligible effects on cell viability compared to untreated cells. Indeed, in all cases, only about 1% of cells were positive to PI staining, just like untreated cells used as control (Figure 3B). Similar analyses performed at 72 h after transfection show that the peptide is not toxic in the concentration range tested up to 72 h (Supplementary Figure S4). On the basis of this evidence, the concentration of the peptide was maintained at 50 μM in all subsequent experiments. MTT experiments were thus performed on cells in the presence of STS at concentrations between 1.0 μM and 20 µM and with AIF(370-394) at 50 μM. Data show that the peptide provided a strong protective effect against cell death induced by the drug at all concentrations tested (Figure 2C). Importantly, up to 10 µM STS cell vitality was fully restored in the presence of peptide. Using STS at 20 µM, more than 75% of cells survived when exposed to AIF(370-394) (Figure 2C). These findings show that treating the cells with the peptide, the pro-apoptotic action of STS is significantly suppressed, and this effect is greater than that observed following silencing of CypA.

### 2.3. AIF(370-394) Influences the AIF/CypA Nuclear Translocation Induced by STS, without Affecting Caspase-3 Activation and PARP

**Figure 4:**
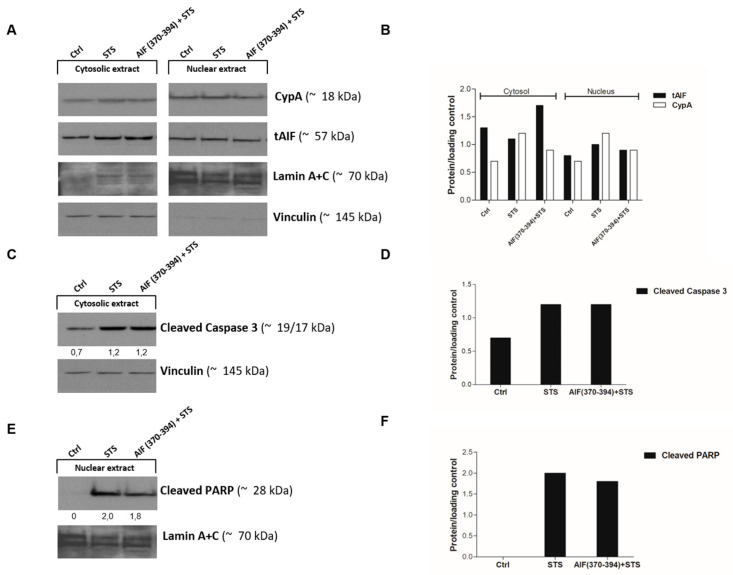
AIF(370-394) blocks AIF and CypA nuclear translocation induced by STS. (**A**) Representative immunoblots of CypA and tAIF and relative densitometric bar graph of proteins (**B**) in the cytosolic and nuclear fractions of SH-SY5Y cells untreated or treated with 5 μM STS for 3 h. (**C**) Representative immunoblot for the detection in the cytosolic extract of cleaved caspase 3 and (**D**) cleaved PARP in the nuclear extract and relative densitometric bar graphs of proteins (**E**). Densitometric analyses were performed using vinculin and lamin A/C as markers of cytosolic and nuclear proteins, respectively.

To investigate the mechanism underlying the protective effects of AIF(370-394) on STS-treated SH-SY5Y cells, we evaluated whether the peptide influenced the subcellular localization of CypA and AIF. SH-SY5Y cells were then transfected with the TAT-conjugated peptide and treated with STS. Peptide-treated cells not exposed to STS were used as controls. Nuclear and cytosolic fractions were extracted and AIF and CypA were detected by WB. As shown in Figure 4, CypA and AIF were stained in both the cytosol and the nucleus of untreated SH-SY5Y cells while, as observed in other cell lines [12,25,26,32], STS treatment induced the translocation of both proteins into the nucleus as a consequence of the kinase inhibitor-induced oxidative stress. Indeed, an increase of the AIF and CypA levels was observed in the nucleus upon STS treatment compared to control cells.

Importantly, in the presence of AIF(370-394), a reduced amount of both proteins was revealed in the nucleus of cells treated with STS as compared to control cells, leading to a significant accumulation of AIF in the cytosol (Figure 4A,B). 

To further elucidate the mechanism underpinning the AIF(370-394) protective effect on the STS-treated SH-SY5Y cells, we also inspected the amounts of cleaved caspase-3 and PARP in both the cytosolic and nuclear extracts of cells treated and untreated with 10 µM STS. According to previous reports [5,34], in STS-treated cells, we detected a significant increase of activated caspase-3 and cleaved PARP, detected as the p24 subunit at ~24 kDa, in the cytosol and nucleus, respectively, compared to control cells (Figure 4C–F). The delivery of AIF(370-394) to STS-treated cells did not alter the levels of cleaved target proteins compared to cells treated with STS alone, indicating that the presence of the peptide did not influence the cell death mediated by caspase activation. Cleaved PARP was unexpectedly not stained in the STS-untreated cells. We hypothesize that at the time point evaluated, the level of cleaved PARP is still too low and is therefore not detected.

## 3. Discussion

Several reports have shown that the nuclear translocation of the AIF/CypA complex is associated with cell death in a variety of different cellular and rodent models of acute brain injury induced by oxidative stress, cerebral hypoxia/ischemia (HI), and even brain trauma. Following HI insults, the complex translocates to cell nuclei where induces chromatin condensation, DNA degradation, and cell death through a caspase-independent mechanism [25,26]. Recently, we have shown that this phenomenon is not restricted to neuronal tissues, but also occurs in in vitro and in vivo models of arrhythmogenic cardiomyopathy [32]. It is noteworthy that blocking the AIF/CypA complex and its nuclear translocation through CypA antisense oligonucleotides and/or the delivery of the inhibitory peptide AIF(370-394) protects against cell death induced by high doses of glutamate in HT22 hippocampal cells, prevents brain injury in neonatal mice undergoing HI, and averts myocyte death during myocardial dysfunction [25,26,32]. Despite the increasing evidence on the crucial role of the AIF/CypA complex in neurological diseases and the importance of its targeting for therapeutic purposes, the role of the AIF/CypA complex in PD has not so far been investigated.

In this framework, we explored the role of the AIF/CypA complex in SH-SY5Y treated with STS, which is a well-known model to study PD in cells [1]. In these cells, it has been demonstrated that, as a consequence of oxidative stress, high doses of STS induce the mitochondrial release of AIF in the cytoplasm and in the nucleus, where it is involved in cell death pathways [7,9,10]. Here, we have demonstrated that, in this experimental paradigm, AIF translocation to the nucleus and the subsequent effects on cell viability requires its association with CypA and that inhibiting the formation of this complex is a way to prevent cell damages induced by oxidative stress.

We found that CypA is highly expressed in this cell line and that treatment with STS increases its levels in both the cytoplasm and the nucleus, very likely due to an inflammatory response, as reported in previous reports [27]. In the same experimental conditions, AIF is also detected in the nucleus, suggesting that the complex AIF/CypA contributes to decreasing cell viability. The dependence of the death mechanism from the AIF/CypA complex formation is strongly supported by the observation that blocking CypA expression with the corresponding antisense significantly neutralizes STS-mediated cell killing and by the protective effects provided by AIF(370-394), which reportedly blocks the nuclear translocation of the complex. Moreover, the overexpression of CypA in SH-SY5Y significantly amplifies the lethal effect of STS while its downregulation strongly counteracts it. Altogether, these data demonstrate the implication of the AIF/CypA complex in the STS-driven mechanism of death of SH-SY5Y cells and that its targeting provides strong neuroprotection.

We have previously demonstrated that blocking the AIF/CypA nuclear translocation with AIF(370-394) not only suppresses apoptosis of HT-22 neuronal cells after glutamate-mediated oxidative stress but also preserves mitochondrial bioenergetics, suggesting alternative pathways of action of the peptide, upstream of lethal nuclear translocation of AIF/CypA [25]. However, this evidence was not confirmed in the model of neonatal mice brain injury after HI [26]. To assess this aspect in SH-SY5Y cells and to determine the impact of the peptide on caspases activation, we also evaluated the activation of caspase-3 upon STS treatment. In SH-SY5Y, STS promotes the permeabilization of the outer mitochondrial membrane [35], inducing the release of several proteins from mitochondria to the cytosol. Cytochrome C is one of the first proteins to translocate into the cytosol, where it activates by an allosteric mechanism, the apoptosis-protease activating factor 1 (APAF-1), which is in turn required for the proteolytic maturation of caspases, including the caspase-3-like proteases [36]. Preliminary experiments show that, as previously reported [36], in SH-SY5Y STS causes a significant increase of the cleaved/activated caspase-3 compared to untreated cells, but interestingly, the presence of the peptide does not appear to affect caspase-3 activation. To further investigate this observation, we have also examined the effect of STS and STS/peptide treatments on PARP cleavage, which is a major hallmark of caspase-3 activation. Consistent with the increase of caspase-3 cleavage, STS induces PARP inactivation [6,7,8], but, in line with previous results, AIF(370-394) does not affect the processing of these proteins in this cell model. These results globally show that inhibiting with AIF(370-394) the AIF/CypA complex in a cellular model of PD prevents cell death and that the mechanism is independent of the caspase pathways and does not affect mitochondrial bioenergetics. However, future experiments are needed to analyze in more detail the pro-apoptotic mechanism mediated by AIF/CypA complex in this experimental paradigm.

The development of effective therapies for PD is extremely challenging because of the limited understanding of the mechanisms of neurodegeneration in PD and the high heterogeneity of the pathology. Peptides are crucial tools for PD research studies and drug discovery [37]. Today several natural and synthetic peptides are used for the treatment of PD, such as Glucagon-like peptide-1 (GLP-1)-based receptor agonists [38] and NAPVSIPQ (NAP) [39]. We thus feel that AIF(370-394) may play a key role for understanding further the mechanisms underlying the disease onset and the potential pathways to target for its treatment, especially those associated with damage of the mitochondrial functions. The successful application of this synthetic peptide highlights the role of the AIF/CypA complex in the pathophysiological mechanisms leading to PD and suggests that it is a promising target for developing first-in-class therapeutics to treat this currently incurable disease. 

## 4. Materials and Methods

### 4.1. Materials

Protected amino acids, coupling agents (HATU, Oxyma), and Fmoc-Rink Amide AM resin used for peptide synthesis were purchased from IRIS Biotech GmbH (Marktrewitz, DE). Solvents, including acetonitrile (CH3CN) and dimethylformamide (DMF) were purchased from Carlo Erba reagents (Milan, Italy). Other products such as trifluoroacetic acid (TFA), sym-collidine, diisopropylethylamine (DIPEA), piperidine, were from Sigma-Aldrich (Milan, Italy). HPLC analyses for peptides characterization were performed on an Alliance HT WATERS 2795 system, equipped with a PDA WATERS detector 2996, whereas preparative purifications were carried out on a WATERS 2545 preparative system (Waters, Milan, Italy) fitted out with a WATERS 2489 UV/Visible detector. 

### 4.2. siRNA

RNA interference experiments were performed as previously described in the literature [25].

### 4.3. Peptide Synthesis and Characterization

AIF(370-394) conjugated at the N-terminus with the cell-penetrating TAT peptide (sequence: GRKKRRQRRRβAFC), which was introduced to favor membrane crossing [40], was assembled on solid phase (Rink-Amide MBHA resin) using a standard protocol for Fmoc chemistry with Oxyma-DIC and HATU-collidine as coupling reagents, as previously reported [41,42]. Peptide purity and identity were confirmed by liquid chromatography–mass spectrometry analysis (LC–MS), as reported in the literature [41,42]. The TAT-conjugated peptide is here called AIF(370-394) for simplicity. A TAT-AIF peptide variant N-terminally modified with fluorescein-5-isothiocyanate (FITC) was also similarly prepared and utilized to assess cell penetration by FACS analyses.

### 4.4. Neuroblastoma SH-SY5Y Cell Culture and Transfection 

SH-SY5Y neuroblastoma cells were obtained from the American Type Culture Collection (ATCC) and cultured in high-glucose DMEM supplemented with 10% FBS (Hyclone), penicillin (100 mg/mL), streptomycin (100 mg/mL), and amphotericin B (250 mg/mL) (Merk Life Science S.r.l. Via Monte Rosa, 93 20149 Milan, Italy). The cells were incubated at 37 °C at a fixed concentration of CO_2_ (5%), and culture medium was changed every 2–3 days.

Transient transfection with siRNA and pEGFP-*cypA* plasmid was performed using Lipofectamine-2000 (Invitrogen) following the manufacturer’s procedure. Peptide transient transfection was performed using the AIF(370-394) peptide conjugated at N-terminus with the TAT peptide [26,32]. Briefly, the peptide was incubated with cells for 4 h in DMEM without serum. After the incubation, culture medium was changed with DMEM supplemented with 10% FBS (Hyclone), penicillin (100 mg/mL), streptomycin (100 mg/mL), and amphotericin B (250 mg/mL) (Sigma, UK).

### 4.5. Cell Treatment

Cells were treated with STS at the indicated times and concentrations. STS was dissolved in DMSO and added to the culture medium to obtain the final concentration indicated. Negative control cells were treated with an equal volume of DMSO (<0.1% *v*/*v*).

### 4.6. Cell Viability Assay (MTT)

The effect of STS on cell viability was determined by the MTT assay (MTT: 3-(4,5-dimethyl thiazol-2yl)-2, 5-diphenyl tetrazolium bromide) [43]. Cells were seeded in a 96-well flat-bottom plate at a density of 6 × 10^3^ cells/well for 24 h at 37 °C in a CO_2_ incubator. After 24 h incubation, the culture medium was replaced with a fresh medium, therefore treated with STS. Subsequently, 10 µL of MTT working solution (5 mg/mL in phosphate buffer solution) were added to each well and the plate was incubated for 4 h at 37 °C in a CO_2_ incubator. The medium was then aspirated, and the formed formazan crystals were solubilized by adding 50 µL of DMSO. Absorbance intensity was measured using an Infinite M200 plate reader (TECAN) at 570 nm. Experiments were performed in triplicate and values are expressed as mean ± SD.

### 4.7. Cell Death Assay by Propidium Iodide (PI)

Cells were plated (2 × 10^5^ cells/mL) and grown for 24 h. Cells were then transfected with the peptide and treated with the STS at the indicated concentration and time. Cell samples were left untreated and used as negative controls. Finally, cells were recovered and incubated with PI buffer containing 0.2 μg/mL of PI in PBS and analyzed by (FACS) calibur flow cytometer using Cell Quest software (Becton Dickinson, BD Biosciences, Drive Franklin Lakes, NJ 07417-1880 USA).

### 4.8. Subcellular Fractionation and Western Blot Analysis

For WB analysis, SH-SY5Y cells were lysed in 50 µL RIPA buffer supplemented with protease inhibitor cocktail and phenylmethylsulphonylfluoride (PMFS) (all from Sigma-Aldrich, Milano, Italy). After centrifugation at 13,000× *g* for 30 min at 4 °C the supernatant was stored at −80 °C until further use. For cytosolic extract preparation, cells were washed in cold PBS, centrifuged at 6000 rpm for 5 min at 4 °C and resuspended in the Cytoplasmic Extract (CE) buffer (10 mM HEPES pH 7.9, 10 mM KCl, 0.1 mM EDTA, 0.3% NP-40 supplemented with protease and phosphatase inhibitors cocktail) on ice for 5 min. After centrifugation at 3000 rpm for 5 min at 4 °C, the supernatant (cytosolic extract) was harvested. The pellet was washed twice with CE buffer without NP-40, centrifuged at 3000 rpm for 5 min at 4 °C, and incubated with an equal volume of Nuclear Extract (NE) buffer (20 mM HEPES, 0.4 M NaCl, 1 mM EDTA, 25% glycerol supplemented with protease inhibitors cocktail) for 10 min. After centrifugation at 14,000 rpm for 5 min at 4 °C the supernatant (nuclear extract) was harvested. The protein concentration was determined by the Bradford assay method. About 30 μg of proteins were separated on 4–12% pre-cast gel (Bolt Bis-Tris Plus, Thermo Fischer, Milano, Italy) followed by transfer to a PVDF membrane. After blocking with 5% skim milk powered in TRIS-buffered saline (TBS)-Tween for 1 h, the membranes were incubated overnight at 4 °C with specific primary antibodies: anti-CypA (GTX 104698, GeneTex, 2456 Alton Pkwy Irvine, CA 92606, USA), anti-Lamin-A/C (GTX 101127, GeneTex, 2456 Alton Pkwy Irvine, CA 92606, USA), anti-AIF (sc 9416, Santa Cruz Biotechnology, Inc. Bergheimer Str. 89-2, 69115 Heidelberg, Germany), anti-caspase 3 (ab32351-Abcam, Prodotti Gianni S.p.A., Via Quintiliano,30, 20138 Milan, Italy), anti-PARP (ab6079-Abcam, Prodotti Gianni S.p.A., Via Quintiliano,30, 20138 Milan, Italy), anti-Vinculin (orb 76294, Biorbyt Ltd., 5 Orwell Furlong Cowley Road Cambridge Cambridgeshire CB4 0WY, UK), and anti-Erk1/2 (sc-514302, Santa Cruz Biotechnology, Inc. Bergheimer Str. 89-2, 69115 Heidelberg, Germany). PVDF membranes were then exposed to the appropriate HRP-conjugated secondary antibody and immunocomplexes were visualized with the ECL detection system (Santa Cruz, CA, USA) and subsequently exposed to film. Relative band intensities were quantified by densitometric analysis with Image J software (NIH, Bethesda, MA, USA).

### 4.9. Statistical Data Analysis

Data were presented as the mean ± SD of biological replicates. Differences in the mean between different groups were calculated using analysis of variance (ANOVA) plus Student’s *t*-test. *p*-values of less than 0.05 were recognized as significant.

## Data Availability

All data are provided as figures and tables and included in this paper.

## Supplementary Materials

The following supporting information can be downloaded at: www.mdpi.com/article/10.3390/ijms23010265/s1.
