# Supplementary material for: Relevance of AIF/CypA Lethal Pathway in SH-SY5Y Cells Treated with Staurosporine
Source: Int J Mol Sci. 2021 Dec 27;23(1):265. doi: 10.3390/ijms23010265 (PMC8745523; doi:10.3390/ijms23010265)
Supplement: Supplementary file 1 [file ijms-23-00265-s001.zip › ijms-1515057-supplementary.pdf]

Supplementary Materials

Communication

Relevance of AIF/CypA lethal pathway in SH-SY5Y cells treated with staurosporine.

Mariarosaria Conte [1], Rosanna Palumbo [2], Alessandra Monti [2], Elisabetta Fontana [1], Angela Nebbioso [1], Menotti Ruvo [2], Lucia Altucci [1,3], and Nunzianna Doti [2,*]

[1] Department of Precision Medicine, University of Campania 'Luigi Vanvitelli', Via L. De Crecchio 7, 80138 Naples, Italy; mariarosaria.conte@unicampania.it (M.C.); elisabetta.fontana@unicampania.it (E.F.); angela.nebbioso@unicampania.it (A.N.); lucia.altucci@unicampania.it (L.A.)

[2] Institute of Biostructures and Bioimaging (IBB), National Research Council (CNR), Via Mezzocannone 16, 80134 Napoli, Italy; rosanna.palumbo@cnr.it (R.P.); alessandra.monti@ibb.cnr.it (A.M.); menotti.ruvo@unina.it (M.R.)

[3] Biogem, Institute of Molecular Biology and Genetics, Via Camporeale Area P.I.P., Ariano Irpino (AV), 83031 Italy; lucia.altucci@unicampania.it (L.A.)

* Correspondence: nunzianna.doti@cnr.it

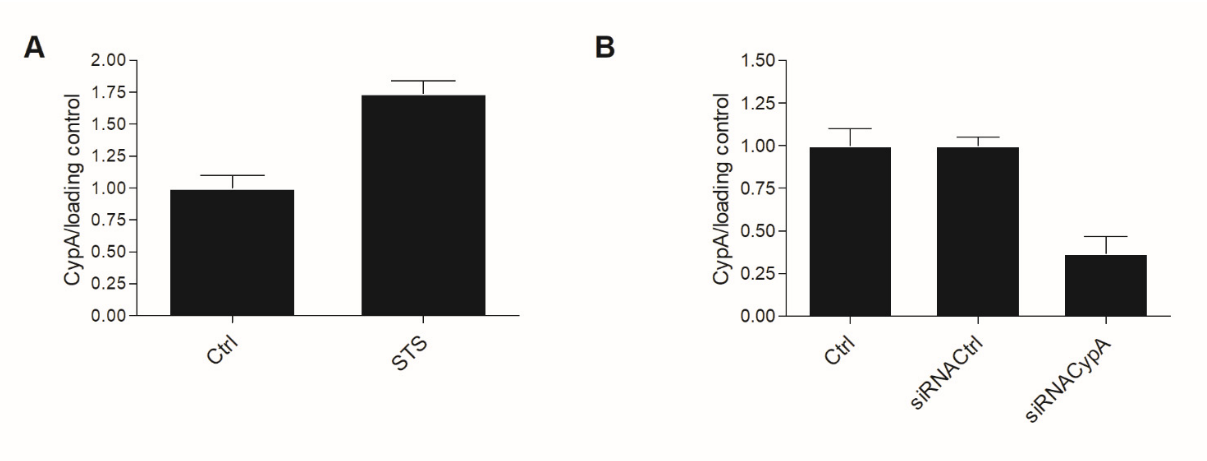

Figure S1: Densitometric analysis of CypA expression levels. Densitometry analysis of CypA protein (A) and CypA protein after transfection of siRNA Ctrl and siRNACypA (B) was performed using ImageJ quantification software to measure the relative band intensity compared to the untreated cells. The final relative quantification values are the ratio of net band to net loading control (ERK1/2). Data represent mean ± standard deviation of three independent experiments.

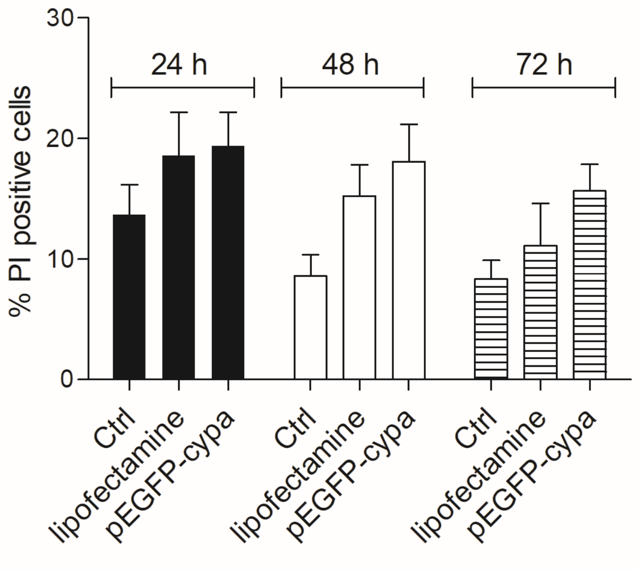

Figure S2: Evaluation of the cytotoxic effect of GFP-fused CypA 24, 48 and 72 h after transfection by FACS analysis; apoptotic cells were stained with PI.

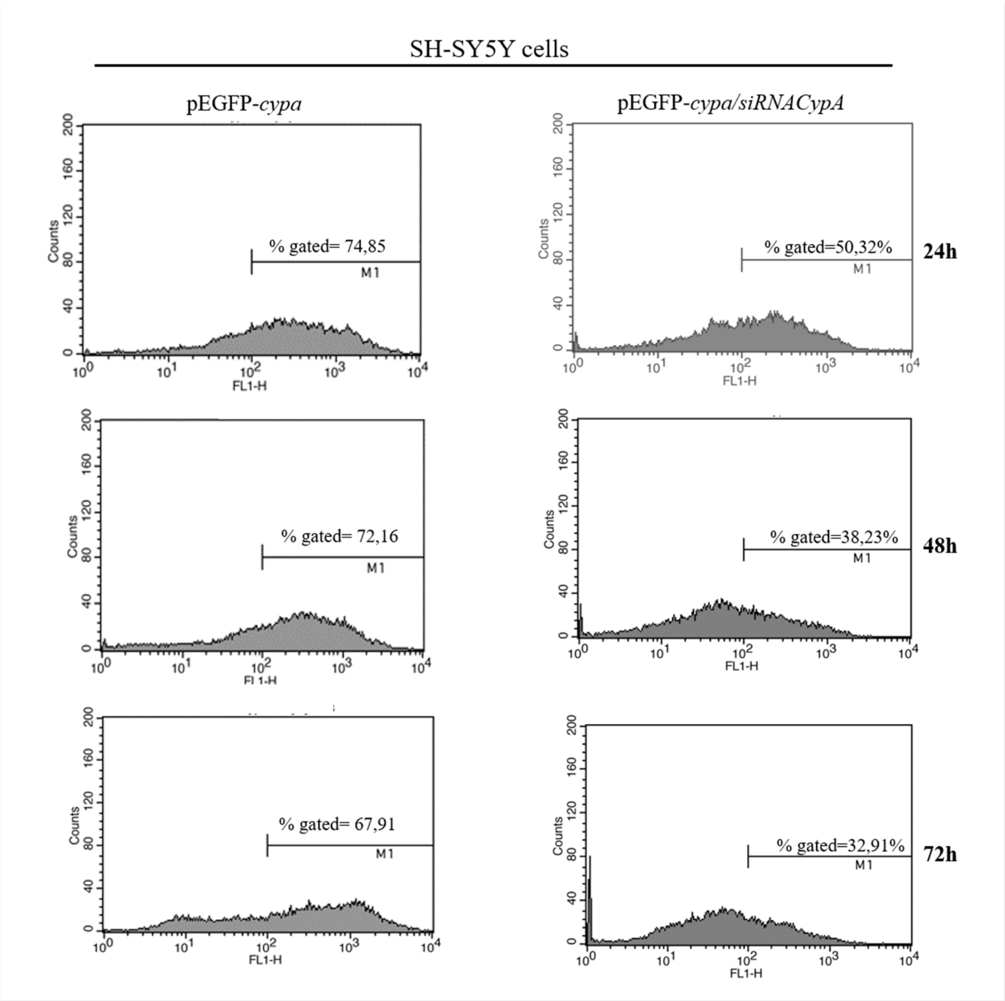

Figure S3: Assessment of CypA downregulation after 24, 48 and 72 h after transfection using by FACS analysis following the GFP fluorescence.

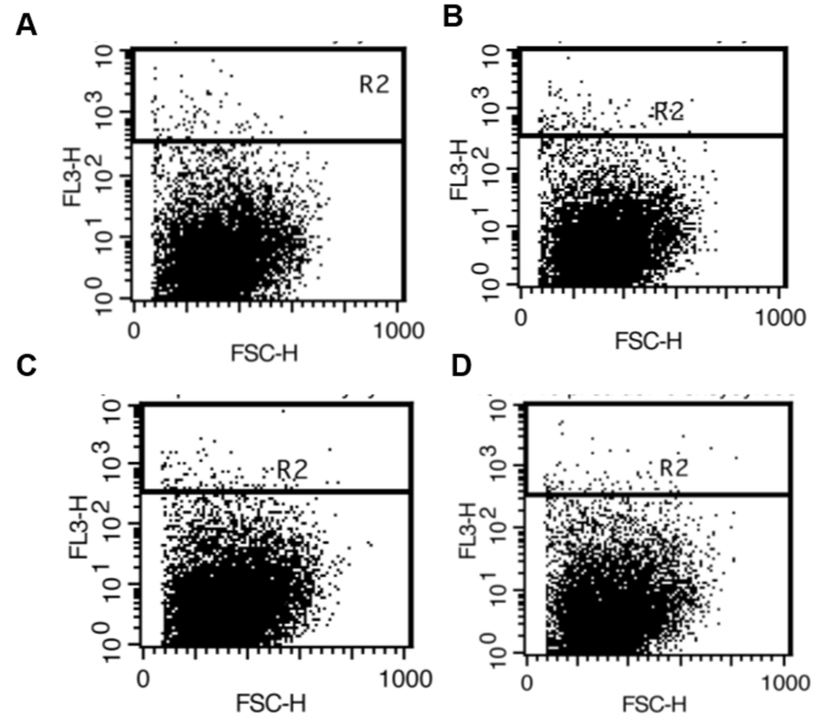

		AIF(370-394)		
	Ctrl	25 µM	50 µM	100 µM
% PI positive cells	0,93	0,98	0,97	0,82

Figure S4: Evaluation of the cytotoxic effect of AIF(370-394) peptide delivery. FACS analysis of untreated cells (A), cells transfected with the peptide at 25 μM (B), 50 μM (C) and 100 μM (D) 72 h after transfection; apoptotic cells were stained with PI.
